# Protective Effects of Myo-Inositol and Selenium on Cadmium-Induced Thyroid Toxicity in Mice

**DOI:** 10.3390/nu12051222

**Published:** 2020-04-26

**Authors:** Salvatore Benvenga, Herbert R. Marini, Antonio Micali, Jose Freni, Giovanni Pallio, Natasha Irrera, Francesco Squadrito, Domenica Altavilla, Alessandro Antonelli, Silvia Martina Ferrari, Poupak Fallahi, Domenico Puzzolo, Letteria Minutoli

**Affiliations:** 1 Department of Clinical and Experimental Medicine, University of Messina, 98125 Messina, Italy; s.benvenga@live.it (S.B.); hrmarini@unime.it (H.R.M.); gpallio@unime.it (G.P.); nirrera@unime.it (N.I.); fsquadrito@unime.it (F.S.); lminutoli@unime.it (L.M.); 2 Department of Biomedical and Dental Sciences and Morphofunctional Imaging, University of Messina, 98125 Messina, Italy; freni.jose@gmail.com (J.F.); daltavilla@unime.it (D.A.); puzzolo@unime.it (D.P.); 3 Department of Clinical and Experimental Medicine, University of Pisa, 56126 Pisa, Italy; alessandro.antonelli@med.unipi.it (A.A.); sm.ferrari@int.med.unipi.it (S.M.F.); poupak.fallahi@unipi.it (P.F.)

**Keywords:** cadmium, nutraceuticals, myo-inositol, seleno-L-methionine, thyroid, MCP-1, CXCL10

## Abstract

Cadmium (Cd) damages the thyroid gland. We evaluated the effects of myo-inositol (MI), seleno-L-methionine (Se) or their combination on the thyroids of mice simultaneously administered with Cd chloride (CdCl_2_). Eighty-four male mice were divided into 12 groups (seven mice each). Six groups (controls) were treated with 0.9% NaCl (vehicle), Se (0.2 mg/kg/day), Se (0.4 mg/kg/day), MI (360 mg/kg/day), MI+Se (0.2 mg/kg) and MI+Se (0.4 mg/kg). The other six groups were treated with CdCl_2_ (2 mg/kg), CdCl_2_+MI, CdCl_2_+Se (0.2 mg/kg), CdCl_2_+Se (0.4 mg/kg), CdCl_2_+MI+Se (0.2 mg/kg) and CdCl_2_+MI+Se (0.4 mg/kg). An additional group of CdCl_2_-challenged animals (n = 7) was treated with resveratrol (20 mg/kg), an effective and potent antioxidant. All treatments lasted 14 days. After sacrifice, the thyroids were evaluated histologically and immunohistochemically. CdCl_2_ reduced the follicular area, increased the epithelial height, stroma, and cells expressing monocyte chemoattractant protein-1 (MCP-1) and C-X-C motif chemokine 10 (CXCL10). CdCl_2_+Se at 0.2/0.4 mg/kg insignificantly reversed the follicular and stromal structure, and significantly decreased the number of MCP-1 and CXCL10-positive cells. CdCl_2_+MI significantly reversed the thyroid structure and further decreased the number of MCP-1 and CXCL10-positive cells. CdCl_2_+MI+Se, at both doses, brought all indices to those of CdCl_2_-untreated mice. MI, particularly in association with Se, defends mice from Cd-induced damage. The efficacy of this combination was greater than that of resveratrol, at least when using the follicular structure as a read-out for a comparison. We suggest that the use of these nutraceuticals, more specifically the combination of MI plus SE, can protect the thyroid of Cd-exposed subjects.

## 1. Introduction

Thyroid disorders, including hypothyroidism, with its leading etiology (Hashimoto’s thyroiditis) and cancer arising from the follicular epithelium, are very common diseases [1,2]. Indeed, with a prevalence of 10%–12% in the general population, Hashimoto’s thyroiditis is the most common autoimmune disease [1]. The prevalence of thyroid cancer has increased considerably in recent decades. 

Thyroid cancer incidence was relatively stable until the 1990s, when it began to increase dramatically. Overall, thyroid cancer incidence increased from 4.9 per 100,000 population to 14.7 per 100,000 population in 2011 [2]. Dysfunction, autoimmunity and cancer of the thyroid are triggered by environmental factors, including pollutants such as organochlorine compounds, polychlorinated biphenyls, polybrominated diphenylethers, bisphenol A, triclosan, perchlorates, thiocyanates, nitrates and heavy metals [3,4,5,6,7,8].

One such heavy metal is cadmium (Cd). Cd is found in food, cigarette smoke, mines, phosphate fertilizers and nickel–cadmium batteries [9]. Cd enters the body through the gastrointestinal tract and the alveolar epithelium [9] and passes through the systemic circulation, bound to albumin. Cd is then transported to the liver, where it is released and induces the synthesis of metallothionein (MT), a stress protein first discovered in 1957 in horse kidneys [10], which protects against Cd toxicity and oxidative stress [11]. The complex MT–Cd accumulates in the liver, kidneys, skeletal muscles and thyroid. With a biological half-life of 5 to 30 years, exposure over time to even environmentally low levels of Cd is associated with several toxic effects on the liver, kidneys, bones, testes and the cardiovascular system [12,13,14,15]. Persons living in Cd-polluted areas have an intrathyroid concentration of Cd that is threefold greater than control persons [16]. Finally, Cd has been classified as a group 1 human carcinogen, with evidence existing of its association with lung, prostate and kidney cancers [17], and a possible association with other malignancies, such as breast [18], pancreas [19], and urinary bladder cancer [20]. Concerning thyroid cancer, a recent study on 66 patients with papillary thyroid cancer (PTC) showed that the content of selenium (Se) was significantly decreased (66 vs. 132 ng/g), while the content of Cd (58 vs. 33 ng/g) and the resulting Cd/Se ratio (0.055 vs. 0.018) were significantly higher in the cancerous tissue compared to the healthy, noncancerous thyroid tissue [21]. Furthermore, Cd and the Cd/Se ratio were associated with the retrosternal thyroid growth of PTC [21].

Cd increases serum thyrotropin (TSH) concentration in rats [22,23] and humans [24]. After chronic exposure to Cd, the presence of desquamated cells in the follicles, mononuclear cell infiltration in the connective tissue and follicles lined by higher cells with light cytoplasm were observed [25]. In a similar manner to mercury (Hg), the interaction of Cd with Se, which is relatively abundant in the thyroid [26], as the inorganic component of the deiodinases [27,28,29], results in formation of insoluble complexes [27,28,30]. The consequence of such sequestration of Se is the impairment of selenoprotein synthesis and activities [28]. In over 5000 Chinese adults, blood levels of both Cd and lead (Pb) were directly correlated with both thyroid hypofunction and serum thyroid autoantibody levels [31], even if the risk of hypothyroidism increased incrementally with blood cadmium in men, but not in women [32]. In the study on 5628 Chinese adults, women showed a positive correlation between log (ln)-transformed blood concentrations of Cd and log (ln)-transformed blood concentrations of thyroglobulin autoantibodies (TgAb) [31]. In the adjusted logistic regression models, the log (ln)-transformed blood concentrations of Cd of women were positively related to their TgAb tertiles and hypothyroid status [31]. Cd exposure also causes increased susceptibility to testicular autoimmunity [33]. Indeed, the role of Cd in autoimmunity is supported by studies on 24 individuals, in whom the authors measured the blood levels of three heavy metals (Cd, Hg and Pb) and blood mRNA expression of 98 genes that are implicated in stress, toxicity, inflammation, and autoimmunity [34].

Among the different mechanisms involved in Cd-induced thyroid damage (genome influence, apoptosis, mitochondrial dysfunction, oxidative stress), the the last mechanism seems to play a relevant role [24]. Indeed, the said depletion of Se stores leads to its decreased availability to form glutathione (GSH) peroxidase, which is one of the main natural antioxidants [35]. Se supplementation exerts some beneficial effects on the thyroid of Cd-exposed rats. Histopathological analysis of the thyroid of young rats whose mothers received Cd during pregnancy demonstrated the presence of microfollicles lined by a single layer of columnar epithelium; Cd administration resulted in a sharp decrease in the height of epithelial cells [36]. Furthermore, treatment of Cd-exposed rats with Se partially attenuated the Cd-induced decrease in serum T4 levels [22].

Beneficial effects against Cd-induced damage of the thyroid or other organs were also described for other naturally occurring molecules that, like Se, are used as nutraceuticals. These molecules are quercetin [37] and myo-inositol (MI) [38,39,40]. Quercetin significantly increased serum thyroid hormones in rats treated with CdCl2, even if levels were significantly lower compared to unchallenged rats [37]. In mice, a 14-day treatment with CdCl_2_ (2 mg/kg/day) plus MI (360 mg/kg/day) protected the testis [39] and the kidney [40] from the damage induced by CdCl_2_. The Cd-induced testicular damage consisted of smaller tubules, the discontinuity of the seminiferous epithelium, the detachment of spermatogonia from the basal membrane, and reduced claudin-11 immunoreactivity [39]. The Cd-induced renal damage consisted of alterations in glomerular and tubular morphology [40]. Concerning the testis, experiments were also conducted with Se alone and with a combination MI plus Se-L-methionine [39], with such a combination having the greatest protective effects on the seminiferous tubules, and in particular on the blood–testis barrier. 

Aside from the Cd setting, the beneficial effects of MI have been shown in human sperm [41,42] and oocytes [43]. The antioxidant properties of MI are demonstrated by the MI-induced increase in the intracellular levels of GSH, superoxide dismutase (SOD) and catalase (CAT) [44]. Starting from the knowledge that chemokines are mechanistically involved in the initiation and maintenance of Hashimoto’s thyroiditis [45,46], it was shown that MI and, to a greater extent, the combination of MI plus Se-L-methionine, decreased serum levels and lymphocyte secretion of the investigated chemokines [47,48]. These chemokines were chemokine (C-C motif) ligand 2 (CCL2; also known as monocyte chemoattractant protein-1 (MCP-1)), C-X-C motif chemokine 9 (CXCL9; also known as monokine induced by gamma interferon (MIG)), and C-X-C motif chemokine 10 (CXCL10). Such effects on chemokines explain the benefits of the combination of MI plus Se-L-methionine in patients with Hashimoto’s thyroiditis, in terms of decreased oxidative stress [38], decreased serum levels of thyroid autoantibodies and an improved hormone profile [47,48,49]. 

Based on this background, considering (i) that no morphometric analysis has been conducted in previous studies on protection from Cd-induced thyroid damage [24,36,50], (ii) the lack of data regarding the effects of MI alone and the association of MI plus Se with Cd-induced thyroid damage, and (iii) that MI, Se and other natural compounds are being increasingly used as nutraceuticals in clinical practice [38], we aimed to demonstrate their protective role in the structure of thyroid glands in mice exposed to Cd. We also wished to test whether Cd induced the expression of two aforementioned chemokines (MCP-1/CCL2 and CXCL10) and, if so, to test whether this expression could be counteracted by MI, Se or their combination.

## 2. Materials and Methods

### 2.1. Experimental Protocol

All procedures adhered to the standards for care and use of animals as per guidelines issued by Animal Research Reporting in Vivo Experiments (ARRIVE); the procedures were evaluated and approved by the Italian Health Ministry (project identification code: 112/2017-PR). Eighty-four male C57 BL/6J mice (25–30 g) were obtained from Charles River Laboratories Italia Srl (Calco, LC, Italy) and stored at the animal house faculty of our university hospital. A standard diet was provided ad libitum and the animals had free access to water; they were kept on a 12-h light/dark cycle. The animals were randomly distributed into twelve groups of seven mice each. Six groups (viz. 42 mice) were considered as controls (0.9% NaCl (vehicle, 1 mL/kg), seleno-L-methionine (Se) (0.2 mg/kg), Se (0.4 mg/kg), MI (360 mg/kg), MI (360 mg/kg) plus Se (0.2 mg/kg), MI (360 mg/kg) plus Se (0.4 mg/kg)). The other six groups were challenged with CdCl_2_ (2 mg/kg) plus a vehicle, CdCl_2_ (2 mg/kg) plus MI (360 mg/kg), CdCl_2_ (2 mg/kg) plus Se (0.2 mg/kg), CdCl_2_ (2 mg/kg) plus Se (0.4 mg/kg), CdCl_2_ (2 mg/kg) plus MI (360 mg/kg) plus Se (0.2 mg/kg) and CdCl_2_ (2 mg/kg) plus MI (360 mg/kg) plus Se (0.4 mg/kg). An additional group of CdCl_2_-challenged animals (*n* = 7) were given 20 mg/kg of resveratrol [51,52], a biologically active compound with potent antioxidant properties [53]. CdCl_2_ and NaCl were administered intraperitoneally (i.p.), while MI, Se and resveratrol per os. MI was ready for use, while CdCl_2_ and Se were diluted in 0.9% NaCl before use. After 14 days of treatment, mice were sacrificed with an overdose of ketamine and xylazine, and their thyroids were collected and processed for histological and immunohistochemical procedures. 

### 2.2. Histological Evaluation

The thyroid glands were fixed in 4% paraformaldehyde in 0.2 M phosphate buffer saline (PBS), dehydrated in ethanol, cleared in xylene and embedded in Paraplast (SPI Supplies, West Chester, PA, USA). Blocks were cut in a microtome (RM2125 RT, Leica Instruments, Nussloch, Germany), and 5 μm sections were cleared with xylene, rehydrated in ethanol and stained with hematoxylin and eosin (HE) and Sirius red (SR). All samples were observed with a Nikon Ci-L (Nikon Instruments, Tokyo, Japan) light microscope and the micrographs were obtained using a digital camera (Nikon Ds-Ri2) and saved as Tagged Image Format Files (TIFF) with the Adobe Photoshop CS5 12.1 software. 

### 2.3. Immunohistochemistry for Monocyte Chemoattractant Protein-1 (MCP-1) and C-X-C Motif Chemokine 10 (CXCL10)

The same specimens used for histological evaluation were cut at 5 μm and the sections were placed on polysine slides (Thermo Fisher Scientific, Waltham, MA, USA), cleared with xylene and rehydrated in ethanol. Antigen retrieval was obtained in buffer citrate pH 6.0; endogenous peroxidase was blocked with 0.3% H_2_O_2_ in PBS. Incubation with primary antibodies (MCP-1, 1:150, Santa Cruz, Dallas, TX, USA; CXCL10, 1:100, Biorbyt, Cambridge, UK) was performed overnight at 4 °C in a moisturized chamber; then, secondary antibodies (anti-mouse and anti-rabbit, Vectastain, Vector, Burlingame, CA, USA) were added, and the reaction was evidenced with 3,3′-diaminobenzidine (DAB) (Sigma-Aldrich, Milan, Italy). Slides were counterstained in Mayer’s hematoxylin. For each test, specific positive and negative controls were prepared. Micrographs were taken with a Nikon Ci-L (Nikon Instruments, Tokyo, Japan) light microscope using a digital camera (Nikon Ds-Ri2).

### 2.4. Morphometric and Immunohistochemical Evaluation

All micrographs were printed at the same final magnification (800×) and were blindly assessed by two trained observers, without knowledge of the previous treatment. Five microscopic fields (MFs), all including two entire thyroid follicles from ten non-serial sections stained with the HE of each group were considered. 

As for the follicular compartment, a Peak Scale Loupe 7× (GWJ Company, Hacienda Heights, CA, USA) micrometer was used as a scale calibration standard to estimate the follicular diameters. The area (A) of each follicle was calculated by measuring the smaller inner diameter (d) and the larger inner diameter (D) of the follicle, both expressed in micrometers (μm). The estimated area of the follicular lumen was obtained by the following formula:

A = π. (d/2. D/2).(1)

In each thyroid gland, we measured the area of 20 follicles. To calculate the epithelial height, a straight line perpendicular to the epithelium was traced and measured, and the results were expressed in micrometers.

For the evaluation of the stroma, a quantitative study of micrographs from 20 microscopic fields of Sirius Red (SR)-stained not-serial sections for each group was performed with the Adobe Photoshop CS5 12.1 software, acquiring the pink/red color of collagen fibers. Positive areas were automatically calculated based on their pixel number. Values were indicated as the pixel number of the positively stained area/unit area (UA). The area of the entire micrograph was evaluated as the UA.

For an assessment of the immunoreactivity of MCP-1/CCL2 and CXCL10, positive cells were counted from 10 non-serial sections of the thyroid, selecting two unit areas (UA) of 0.1 mm^2^ (316 × 316 μm). Cells overlapping the right and top borders of the areas were not counted, while the cells overlapping the left and the bottom borders were considered.

### 2.5. Drugs and Chemicals

CdCl_2_, Se and resveratrol were bought from Sigma-Aldrich Srl (Milan, Italy). LO.LI. Pharma Srl (Rome, Italy) kindly provided MI. All chemicals not otherwise mentioned were commercially available reagent grade quality. 

### 2.6. Statistical Analysis

Values are expressed as the mean ± standard error (SE). The statistical significance of differences between group mean values was established using Student’s t-test. The statistical evaluation of differences among groups was obtained with ANOVA. The statistical analysis of histological scores was done using the Mann–Whitney U test with Bonferroni correction. A *p* value of ≤ 0.05 was considered statistically significant.

## 3. Results

### 3.1. Histopathological Data

#### 3.1.1. Follicular Epithelium

**Figure 1:**
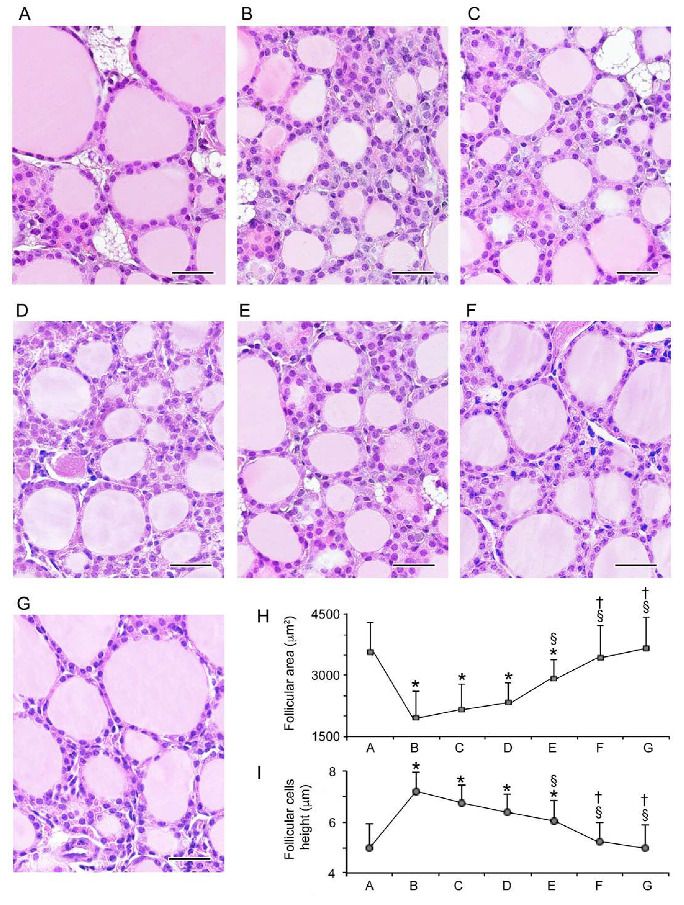
Histological organization of the thyroid (hematoxylin–eosin stain; scale bar: 25 µm). Mice groups (seven mice/group) are: controls (**A**), cadmium chloride (CdCl_2_) plus vehicle (**B**), CdCl_2_ plus seleno-L-methionine (Se) 0.2 mg/kg (**C**), CdCl_2_ plus Se 0.4 mg/kg (**D**), CdCl_2_ plus myo-inositol (MI) (**E**), CdCl_2_ plus MI plus Se 0.2 mg/kg (**F**), CdCl_2_ plus MI plus Se 0.4 mg/kg (**G**). A: Control mice have a normal thyroid structure, as demonstrated also by bar A in H and I. B: CdCl_2_-treated mice show small follicles and less stainable follicular epithelium (thyrocytes), the height of which is increased, as shown by bar B in (**H**,**I**). C-D: In mice treated with CdCl_2_ plus 0.2 mg/Kg Se or CdCl_2_ plus 0.4 mg/kg Se, small follicles are present with thyrocytes of smaller height, as indicated by bars C and D in H and I. E: In mice treated with CdCl_2_ plus MI, the follicles and thyrocytes show a tendency to acquire a normal size and height, even though both indices are significantly different from the controls; see also bar E in H and I. F-G: In mice treated with CdCl_2_ plus MI and 0.2 mg/Kg Se or CdCl_2_ plus MI and 0.4 mg/kg Se, follicles and epithelial cells were close to normal, as demonstrated by bars F and G in H and I. H: Mean ± standard error values of follicular area in the different groups of mice. I: Mean ± standard error values of epithelial cells height in the different groups of mice. * *p* < 0.05 versus control; § *p* < 0.05 versus CdCl_2_ plus vehicle and CdCl_2_ plus 0.2 or 0.4 mg/kg Se; † *p* < 0.05 versus CdCl_2_ plus MI alone.

Images are presented in Figure 1A–G, with the quantification of the follicular area and the height of the follicular epithelium (thyrocytes) summarized in Figure 1H–I. 

All control animals had thyroids with normal morphologies (results not shown). Therefore, for sake of simplicity, we present a single micrograph as representative of controls (Figure 1A). In the thyroid of mice challenged with CdCl_2_, compared to controls, the follicular area was smaller and thyrocytes were taller but poorly stained (Figure 1B). In the thyroid of mice treated with CdCl_2_ plus either dose of Se, compared to controls, the follicular area was also dose-dependently significantly smaller and the follicular epithelium was also dose-dependently significantly taller (Figure 1C,D), though the area and height changed to a lesser degree compared to mice treated with CdCl_2_ alone (compare Figure 1C,D with Figure 1B, and corresponding points in Figure 1H,I). In the thyroid of mice treated with CdCl_2_ plus MI, compared to controls, the follicular area was also significantly smaller, and the follicular epithelium was significantly taller (Figure 1E), while both the area and height changed to an even lesser degree compared to mice treated with CdCl_2_ plus either dose of Se (compare Figure 1E with Figure 1B, and corresponding points in Figure 1H,I). In mice treated with CdCl_2_ plus both MI and Se at either 0.2 or 0.4 mg/kg, the follicular area and epithelial height were no longer statistically different from the controls (Figure 1F,G), but both indices were statistically different from their counterparts in the other treated mice (compare Figure 1F,G with Figure 1B,G, and corresponding points in Figure 1H,I). Furthermore, the effect of the combination of MI plus Se on the follicular area and epithelial height was greater than that brought about by resveratrol, used for its potent antioxidant properties (see Supplemental materials Figure S1).

#### 3.1.2. Stroma

**Figure 2:**
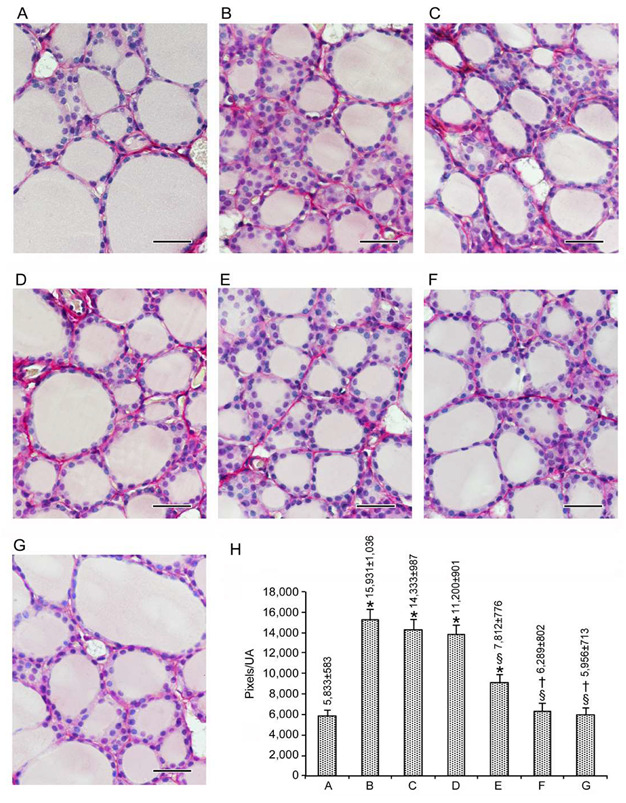
Structural organization of the thyroid stroma based on staining with Sirius Red (scale bar: 25 µm). Mice groups are as in Figure 1. (**A**): Control mice have a normal architecture of interstitial collagen, with well-stained fibrillary elements, as indicated by bar A in H. (**B**): In CdCl_2_-treated mice, an increased amount of perifollicular connective tissue is present around the follicles, as indicated by bar B in H. (**C**,**D**): In mice challenged with CdCl_2_ and 0.2 mg/kg Se or CdCl_2_ and 0.4 mg/kg Se, stained areas are similar to mice challenged with CdCl_2_, as shown by bars C and D in H. (**E**): CdCl_2_ plus MI-treated mice have stained areas with a statistically significant decrease in the pink/red colored collagen fibers, as evident in H, bar E. (**F**,**G**): In mice treated with CdCl_2_ plus MI and 0.2 mg/kg Se or CdCl_2_ plus MI and 0.4 mg/kg Se, a significant reduction in the stained areas can be seen, as also demonstrated by bars F and G in H. (**H**): Mean ± standard error values of pixel numbers of Sirius Red (SR)-stained areas/unit areas (UA) in the different groups of challenged mice. * *p* < 0.05 versus control; § *p* < 0.05 versus CdCl_2_ plus vehicle and CdCl_2_ plus 0.2 or 0.4 mg/kg Se; † *p* < 0.05 versus CdCl_2_ plus MI.

As indicated in the Materials and Methods, Sirius Red staining allowed us to quantify the thyroid stroma, since stromal areas are positive to such staining. Positive areas were quantified by a software based on their pixel number. Data were expressed as the pixel number of the positively stained area/unit area (UA), considered to be the entire micrograph area. Matching the illustration of data for the epithelium (see above), images are presented in Figure 2A–G, with quantification also summarized in Figure 2H.

When comparing Figure 2H with Figure 1H,I, it is evident that changes in the stroma were parallel to changes in epithelial height and opposite to changes in follicular area. Compared to the amount of stroma in the seven untreated mice (5912 ± 556 pixels/UA (data not shown)), in the other 42 control mice, the variation was between −5.0% (mice treated with only 0.2 mg/kg Se) AND +2.1% (mice treated with vehicle) (data not shown). Accordingly, the value of 5833 ± 583 (mean ± SE of the 49 control mice; Figure 2A and bar A in Figure 2H) was taken as the reference to evaluate the effects of CdCl_2_ alone or CdCl_2_ co-administered with Se, MI, or their association. Exposure to CdCl_2_ alone increased the amount of perifollicular connective tissue by almost threefold (compare Figure 2A,B, and bars A and B in Figure 2H). The co-administration of CdCl_2_ and Se decreased—even though the CdCl_2_-induced an insignificant increase in—thyroid connective tissue (compare Figure 2C,D with Figure 2B, and bars C and D with bar B in Figure 2H). MI co-administration was more effective compared to either dose of Se, since the increase in the amount of stroma was of a smaller magnitude (compare Figure 2E with Figure 2C,D, and bar E with bars C-D in Figure 2H) or approximately 1.5-fold higher when compared to controls (compare Figure 2E with Figure 2A, and bar E with bar A in Figure 2H). The association of MI with either dose of Se in mice that were simultaneously treated with CdCl_2_ was even more effective, resulting in an amount of stroma superimposable to that of the control mice (compare Figure 2F,G with Figure 2A, and bars F-G with bar A in Figure 2H).

In summary, both the follicular epithelium and the connective tissue respond in a similar fashion to the administration of Se, MI or their combination in CdCl_2_ co-treated mice. This response consists of a benefit that is modest in the case of Se, moderate in the case of MI, and high (full protection) in the case of MI combined with either dose of Se.

### 3.2. Immunohistochemical Expression of MCP-1/CCL2

**Figure 3:**
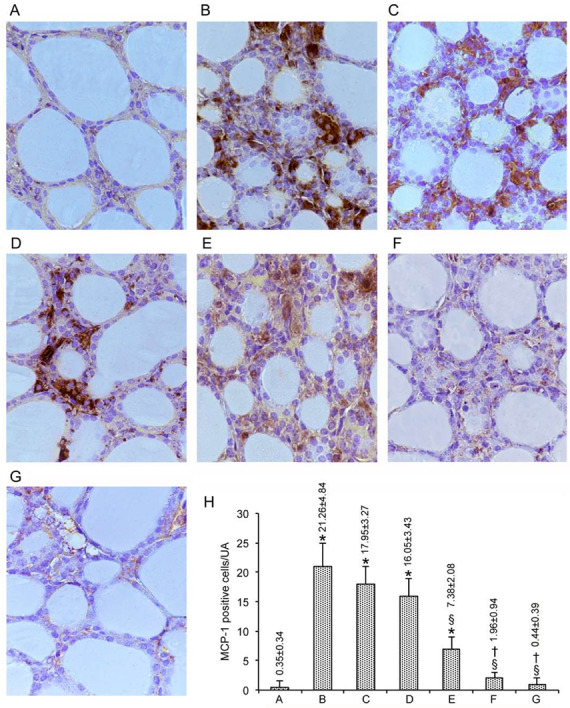
Immunohistochemical expression of monocyte chemoattractant protein-1 (MCP-1) in the thyroid (scale bar: 25 µm). Mice groups are as in Figure 1. (**A**): In controls, no MCP-1-positive cells are present, as shown by bar A in H. (**B**): CdCl_2_-treated mice show a marked increase in MCP-1 immunoreactivity, as indicated by bar B in H; positive cells line the follicle wall with strong stains on their cytoplasm. (**C**,**D**): In mice treated with CdCl_2_ plus 0.2 mg/kg Se or CdCl_2_ plus 0.4 mg/kg Se, the number of MCP-1-positive cells is decreased, but still higher than controls, as evidenced by bars C and D in H. (**E**): CdCl_2_ plus MI-treated mice, MCP-1-positive cells are fewer, as shown by bar E in H, and show reduced cytoplasmic staining. (**F**,**G**): In the thyroid of mice treated with CdCl_2_ plus MI and 0.2 mg/Kg Se or CdCl_2_ plus MI and 0.4 mg/kg Se, MCP-1 immunoreactivity is significantly decreased, as indicated by bars F-G in H. (**H**): The number of MCP-1-positive cells per microscopic field in the different groups of mice (mean ± standard error). * *p* < 0.05 versus control; § *p* < 0.05 versus CdCl_2_ plus vehicle and CdCl_2_ plus 0.2 or 0.4 mg/kg Se; † *p* < 0.05 versus CdCl_2_ plus MI.

**Figure 4:**
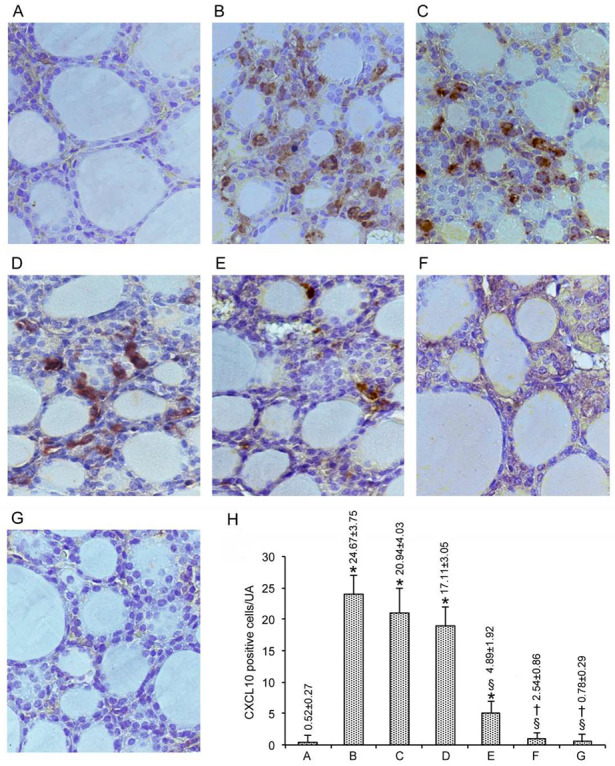
Immunohistochemical expression of C-X-C motif chemokine 10 (CXCL10) in the thyroid (scale bar: 25 µm). Mice groups are as in Figure 1. (**A**): In controls, no CXCL10-positive cells are evident, as shown by bar A in H. (**B**): In CdCl_2_-treated mice, a large number of CXCL10-positive cells is present among the follicle walls, as indicated by bar B in H. (**C**,**D**): In mice treated with CdCl_2_ plus 0.2 mg/kg Se or CdCl_2_ plus 0.4 mg/kg Se, the number of CXCL10-positive cells is decreased, but still high, as evidenced by bars C and D in H. (**E**): In CdCl_2_ plus MI-treated mice, only a few CXCL10-positive cells are present, as shown by bar E in H. (**F**,**G**): In the thyroid of CdCl_2_ plus MI and 0.2 mg/kg Se or CdCl_2_ plus MI and 0.4 mg/kg Se, no CXCL10 immunoreactivity is detectable, as indicated by bars F-G in H. (**H**): The number of CXCL10-positive cells per microscopic field in the different groups of mice (mean ± standard error).* *p* < 0.05 versus control; § *p* < 0.05 versus CdCl_2_ plus vehicle and CdCl_2_ plus Se 0.2 or 0.4 mg/kg Se; † *p* < 0.05 versus CdCl_2_ plus MI.

To maintain the modality of illustrating results, images are presented in Figure 3A–G, with quantification also summarized in Figure 3H for MCP-1/CCL2, and in Figure 4A–G, with quantification also summarized in Figure 4H for CXCL10.

In the baseline condition represented by untreated mice, there were no cells at all that expressed MCP-1/CCL2 (data not shown), a pattern that was also true for the vehicle-treated mice and the other groups of control mice, except three groups. These three groups, in which only one cell was stained, were those treated with either concentrations of Se alone and the group treated with 0.4 mg/kg Se plus MI (data not shown). Overall, in the 49 mice from the seven control groups, the number of cells immunostained by the MCP-1/CCL2 averaged 0.35 ± 0.34/UA (Figure 3A, and bar A in Figure 3H). In contrast, CdCl_2_ plus the vehicle induced a marked expression of MCP-1/CCL2, with a 60-fold increase in the number of thyrocytes immunostained (compare Figure 3B with Figure 3A, and bar B with bar A in Figure 3H). In the mice from the remaining groups, the over-expression induced by CdCl_2_ was counteracted significantly by all of the tested compounds (Figure 3C–G, and bars C-G in Figure 3H). This antagonism was small with either the dose of Se alone (Figure 3C,D, and bars C-D in Figure 3H), moderate with MI alone (Figure 3E, and bar E in Figure 3H), and great with the dose of Se and MI (Figure 3F–G, and bars F-G in Figure 3H). Indeed, 0.4 mg/kg Se plus MI decreased the number of cells to 0.44 ± 0.39, which is statistically similar to the above 0.35 ± 0.34 for the 49 control mice.

### 3.3. Immunohistochemical Expression of CXCL10

When CXCL10 immunoreactivity was considered, the results mimicked those described above for MCP-1/CCL2. Thus, in the 49 mice from the seven control groups, the number of cells immunostained by the CXCL10 averaged 0.52 ± 0.27/UA (Figure 4A, and bar A in Figure 4H). The overexpression of CXLC10 induced by CdCl_2_ plus the vehicle was fully counteracted by the combination of MI with either dose of Se (Figure 4F,G, and bars F-G in Figure 4H).

## 4. Discussion

In the present work, we have confirmed that Cd exposure has negative consequences for the murine thyroid. These consist of histologically demonstrable alterations in the follicular epithelium and stroma, and in the induced expression of two chemoattractant chemokines, an expression that is absent prior to Cd exposure.

In fact, after chronic exposure to Cd, desquamated cells into the follicles, mononuclear cell infiltration in the stroma and follicles lined by higher cells with light cytoplasm were observed [25]. Therefore, these Cd-elicited thyroid changes have consequences in terms of both thyroid dysfunction and autoimmunity [31,32,54].

Se is considered to exert an overall protection against toxicity induced by heavy metals such as Cd, Pb, As and Hg [55], mainly through the sequestration of these elements into biologically inert complexes and/or through the action of Se-dependent antioxidant enzymes [55]. This protection from Cd toxicity occurs regardless of the Se form (as selenite, selenomethionine, nanoSe, or Se from lentils) [56]. Furthermore, it was recently demonstrated that Se alleviated oxidative stress in chicken ovari and rat kidneys, and counteracted the endoplasmic reticulum stress able to induce apoptosis [57,58]. However, in this paper, we found that the trace element Se was less potent than the carbocyclic sugar MI in protecting mice from CdCl_2_ thyroid toxicity, though the co-administration of Se amplified the protection conferred by MI alone. The complementary activity of the two antioxidants can be related to their distinctive mechanism of action. While Se is a vital constituent of the enzyme glutathione peroxidase that catalyzes the reaction between GSH and hydrogen peroxide, thus protecting against oxidative stress, MI is a hydroxyl radical scavenger, preventing lipid peroxidation. The combined action of the two compounds may enhance the antioxidant effect [39]. Interestingly the efficacy of this combination was greater that that of resveratrol (used for its potent antioxidant properties), at least when using the follicular area and the epithelial height as read-outs for a comparison evaluation.

One limitation of our work is the lack of hormone measurements. However, considering (i) the aforementioned Chinese study on the direct relationship between blood Cd levels with thyroid hypofunction and serum thyroid autoantibody levels [31], (ii) the association of insulin resistance with either decreased thyroid hormone levels or increased serum TSH [59,60,61], with counteracting effects by insulin-sensitizing agents [62,63,64], and (iii) the insulin-mimetic action of MI [54,65,66], we expected that Cd-exposed mouse thyroids would display decreased thyroid hormone levels and increased TSH compared to Cd-unexposed mice. We also expected that at least the combination of MI+Se would have fully counteracted these hormone changes induced by Cd.

On the other hand, the strengths of this study are the findings that are consistent with the previous literature concerning the benefits for the thyroid [38] and other endocrine organs, such as the testes [39], including consistency in the hierarchy of benefits (MI+Se > MI >> Se). In particular, CdCl_2_ determined significant increase in MCP-1 and CXCL10-positive cell numbers. Our data clearly agree with many in vitro and in vivo experiments by different groups, showing that the production of these chemokines by thyrocytes may play a central role in the recruitment of monocytes and T-lymphocytes at immune inflammatory sites in the thyroid gland from the blood in humans, thus providing a possible mechanism by which thyrocytes themselves may participate in the processes of thyroid autoimmune and inflammatory disease [45,46]. After treatment with Se, MCP-1 and CXCL10-positive cell numbers were reduced. These data about the effects of Se on the thyroid gland, from our point of view, are not particularly surprising. In fact, the recent literature indicates that, regarding thyroid pathology, selenium intake has been associated with autoimmune disorders [67]. Our experimental data indicate the relative inefficacy of Se when administered alone, probably due to extensive detrimental effects of Cd on thyroid structure and function, not adequately counterbalanced by micronutrient administration alone. MI treatment significantly lowered MCP-1 and CXCL10-positive cell numbers, particularly in association with Se, thus confirming that this nutraceutical compound could impact different molecular pathways related to oxidative stress and inflammation, involving chemokines such as MCP-1 and CXCL10 [46].

In view of the “prophylactic” benefit reported in the present paper, it will now be interesting to investigate whether Se, MI and their combination (Se+MI) have “therapeutic” benefits. The demonstration of the latter’s benefits requires that animals would be first exposed to CdCl_2_ for a time sufficient to induce thyroid toxicity (14 days, based on the present work), and then administered Se, MI and Se+MI at doses equal to or greater than those used in the present work and for the same or a longer time (≥14 days), in order to show the reversal of the alterations induced by prior Cd exposure. Another translational implication of the data presented in this paper is that, because several pollutants, such as organochlorine compounds, polychlorinated biphenyls, polybrominated diphenylethers, bisphenol A, triclosan, perchlorates, thiocyanates, nitrates and heavy metals different from Cd [3,4,5,6,7,8] disturb thyroid homeostasis and confer increased environmental susceptibility to thyroid autoimmunity, it would be worthwhile to test whether the said molecules (particularly MI+Se) may have prophylactic and/or therapeutic effects against the thyroid alterations caused by exposure to a number of thyroid-disrupting chemicals.

## Supplementary Materials

The following are available online at https://www.mdpi.com/2072-6643/12/5/1222/s1, Figure S1: Histological organization of the thyroid (hematoxylin–eosin stain; scale bar: 25 µm). Mice groups (7 mice/group) are: (A) controls, (B) cadmium chloride (CdCl_2_) plus vehicle, (C) CdCl_2_ plus seleno-L-methionine (Se) 0.2 mg/kg, CdCl_2_ plus Se 0.4 mg/kg, (E) CdCl_2_ plus myo-inositol (MI), (F) CdCl_2_ plus MI plus Se 0.2 mg/kg, CdCl_2_ plus MI plus Se 0.4 mg/kg. A: Control mice have normal thyroid structure, as demonstrated also by bar A in I and L. B: CdCl_2_-treated mice show small follicles and less stainable follicular epithelium (thyrocytes), the height of which is increased, as shown by bar B in I and L. C-D: In mice treated with CdCl_2_ plus 0.2 mg/Kg Se or CdCl_2_ plus 0.4 mg/kg Se, small follicles are present with thyrocytes of smaller height, as indicated by bars C and D in I and L. E: In mice treated with CdCl_2_ plus MI, the follicles and thyrocytes show a tendency to acquire the normal size and height, even though both indices are significantly different from controls; see also bar E in I and L. F-G: In mice treated with CdCl_2_ + MI + 0.2 mg/Kg Se or CdCl_2_ + MI + 0.4 mg/kg Se, follicles and the epithelial cells were close to normal, as demonstrated by bars F and G in in I and L. H: In mice treated with CdCl_2_ plus resveratrol, the follicles show an increased size and thyrocytes a reduced height, being both indices significantly different from controls, as shown also by bar H in I and L histograms. I - Mean ± standard error values of follicular area in the different groups of mice. L - Mean ± standard error values of epithelial cells height in the different groups of mice. * *p* < 0.05 versus control; § *p*< 0.05 versus CdCl_2_ plus vehicle and CdCl2 plus 0.2 or 0.4 mg/kg Se; † *p* < 0.05 versus CdCl_2_ plus MI alone; ≠ *p* < 0.05 versus CdCl_2_ + MI + 0.2 mg/Kg Se and CdCl_2_ + MI + 0.4 mg/kg Se.
