# Supplementary material for: Protective Effects of Myo-Inositol and Selenium on Cadmium-Induced Thyroid Toxicity in Mice
Source: Nutrients. 2020 Apr 26;12(5):1222. doi: 10.3390/nu12051222 (PMC7282027; doi:10.3390/nu12051222)
Supplement: Supplementary file 1 [file nutrients-12-01222-s001.pdf]

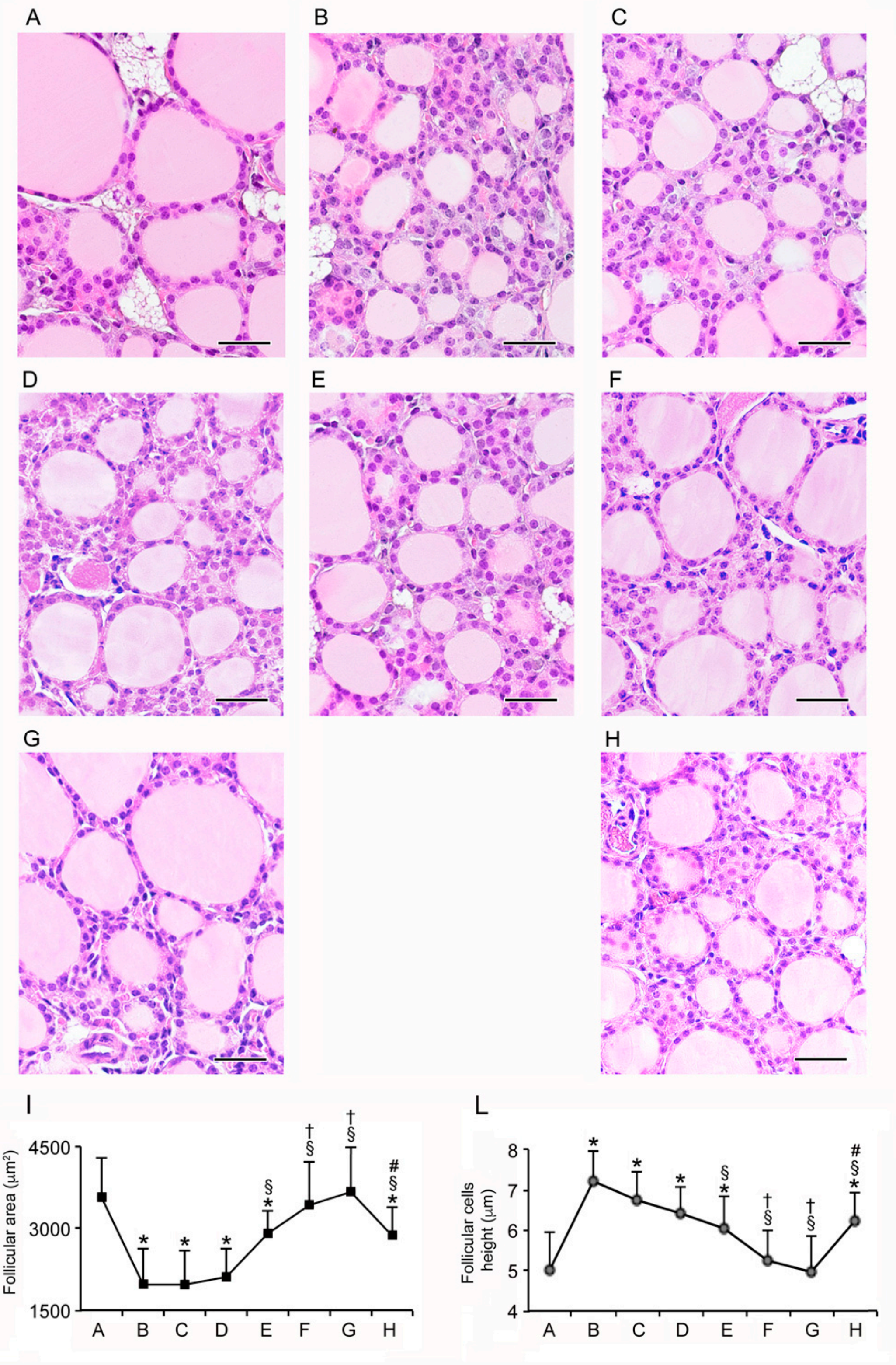

Supplemental figure

Supplemental figure legend - Histological organization of the thyroid (Hematoxylin-Eosin stain - Scale bar: 25 μm). Mice groups (7 mice/group) are: controls, $CdCl_2$ plus vehicle, $CdCl_2$ plus Se 0.2 mg/kg, $CdCl_2$ plus Se 0.4 mg/kg, $CdCl_2$ plus MI, $CdCl_2$ plus MI plus Se 0.2 mg/kg, $CdCl_2$ plus MI plus Se 0.4 mg/kg, resveratrol (20 mg/kg). A: Control mice have normal thyroid structure, as demonstrated also by bar A in I and L. B: $CdCl_2$-treated mice show small follicles and less stainable follicular epithelium (thyrocytes), the height of which is increased, as shown by bar B in I and L. C-D: In mice treated with $CdCl_2$ plus 0.2 mg/Kg Se or $CdCl_2$ plus 0.4 mg/kg Se, small follicles are present with thyrocytes of smaller height, as indicated by bars C and D in I and L. E: In mice treated with $CdCl_2$ plus MI, the follicles and thyrocytes show a tendency to acquire the normal size and height, even though both indices are significantly different from controls; see also bar E in I and L. F-G: In mice treated with $CdCl_2$ + MI + 0.2 mg/Kg Se or $CdCl_2$ + MI + 0.4 mg/kg Se, follicles and the epithelial cells were close to normal, as demonstrated by bars F and G in in I and L. H: In mice treated with $CdCl_2$ plus resveratrol, the follicles show an increased size and thyrocytes a reduced height, being both indices significantly different from controls, as shown also by bar H in I and L histograms. I - Mean ± standard error values of follicular area in the different groups of mice. L - Mean ± standard error values of epithelial cells height in the different groups of mice.

* $p < 0.05$ versus control; § $p< 0.05$ versus $CdCl_2$ plus vehicle and $CdCl_2$ plus 0.2 or 0.4 mg/kg Se; † $p< 0.05$ versus $CdCl_2$ plus MI alone; ≠ $p< 0.05$ versus $CdCl_2$ + MI + 0.2 mg/Kg Se and $CdCl_2$ + MI + 0.4 mg/kg Se.
